# Silk Bioprotein as a Novel Surgical-Site Wound Dressing: A Prospective, Randomized, Single-Blinded, Superiority Clinical Trial

**DOI:** 10.1093/asjof/ojad071

**Published:** 2023-10-20

**Authors:** Daniel S Rouhani, Navin K Singh, James J Chao, Adah Almutairi, Rebecca Badowski-Platz, Mehran H Seradj, Mehrdad Mark Mofid

## Abstract

**Background:**

Medical adhesive-related skin injuries (MARSIs) affect about 1.5 million patients annually in the United States. Complications include allergic contact dermatitis, skin blistering, skin tears, and surgical-site infections (SSIs). The authors hypothesize that a natural hypoallergenic silk bioprotein wound dressing will decrease the incidence of MARSI in comparison to a synthetic alternative.

**Objectives:**

This study aimed to assess the efficacy and safety of a silk bioprotein wound dressing compared to the Dermabond Prineo (Ethicon, Inc., Somerville, NJ) skin closure system.

**Methods:**

This prospective, randomized, single-blinded trial studied 25 patients who were dressed with Dermabond Prineo on one side of their body and on the contralateral side with the silk bioprotein dressing after undergoing abdominoplasty or reduction mammaplasty procedures. Data were collected over 5 postoperative visits using photographs and an investigator administered questionnaire to track rash, itch, discomfort, erythema, edema, SSIs, need for pharmaceutical intervention, mechanical injury, removal time, and bathing routines.

**Results:**

Sixty-four percent (16/25) of patients characterized the severity of discomfort as a score of 4 out of 10 or greater on the Dermabond Prineo control side and only 4% (1/25) for the silk-dressing side (*P* < .001). Fifty-two percent (13/25) had a visible rash of 4 or higher on the Dermabond Prineo side of their incision and 0% (0/25) had a rash on the silk side (*P* < .001). Fifty-two percent (13/25) required steroids or antibiotics to treat MARSI to Dermabond Prineo and 0% (0/25) required pharmaceutical intervention on the silk side (*P* < .001).

**Conclusions:**

The use of a silk bioprotein wound dressing significantly reduces the incidence of MARSI throughout the postoperative period.

**Level of Evidence: 2:**

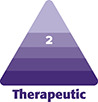

Medical adhesive-related skin injuries (MARSIs) affect approximately 1.5 million patients annually in the United States alone, yet remain one of the most overlooked complications of surgery.^1^ MARSI results from the use of surgical tapes, dressings, and tissue adhesives which can lead to a range of complications including allergic contact dermatitis (ACD), wound dehiscence, surgical-site infections (SSIs), and in extreme cases, sepsis leading to death.^2,3^ A survey of 918 wound-care health professionals in 2016 found that 71% of respondents stated that the occurrence of MARSI was not reported in their facility, and only 31% were aware of the term MARSI.^4^ In addition, a cross-sectional study of 143 spinal surgery patients in 2021 found that MARSI affects up to 36.4% of patients in surgical settings.^5^ This study also identified one of the most common types of MARSI as ACD, a Type IV hypersensitivity reaction that can lead to pruritis, erythema, edema, wound dehiscence, and severe pain.^6^

ACD to the liquid skin adhesive Dermabond (Ethicon, Inc., Somerville, NJ) has been increasingly reported after orthopedic, abdominal, and breast surgeries (Supplemental Figures 1, 2).^7-12^ Dermabond contains 2-octyl cyanoacrylate which polymerizes in the presence of moisture to form a solid adhesive. Dermabond is often applied to the synthetic polyester mesh Prineo (Ethicon, Inc.), collectively known as the Dermabond Prineo Skin Closure System. The Food and Drug Administration's (FDA) Manufacturer and User Facility Device Experience (MAUDE) database has documented 1252 adverse events associated with the use of Dermabond Prineo in the past 9 years. Approximately 75% of these events (939/1252) have been associated with ACD, including reports of hypersensitivity reactions and erythema. Reports of mechanical skin tears and blisters have also been recorded. These complications can damage epidermal cells, which compromise the skin's protective barrier, thus increasing the susceptibility of the wound to SSIs (Figure 1).^1,7-11^ Due to the underreporting of adverse events to the FDA, the presented MAUDE data provide only a limited insight into the actual incidence of MARSI with Dermabond Prineo.^13^

**Figure 1.**
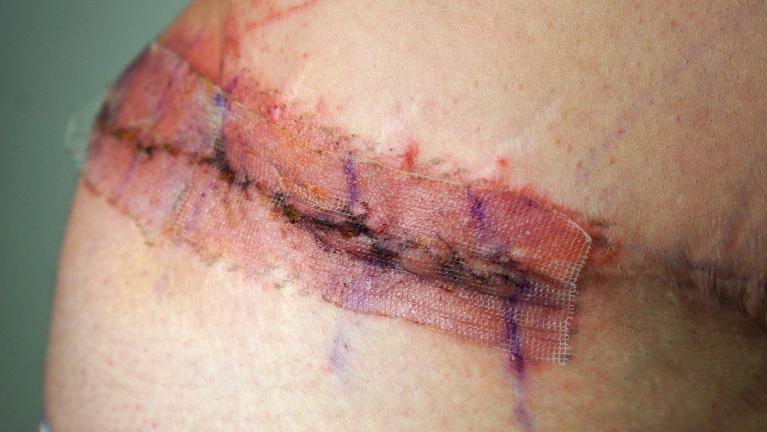
An example of allergic contact dermatitis to Dermabond Prineo (Ethicon, Inc.) following revision abdominoplasty. A 40-year-old female developed a pruritic rash localized in the rectangular region of the Prineo polyester mesh.

In this study, we propose the first indication of use for a silk bioprotein wound dressing to reduce the incidence of MARSI compared to the Dermabond Prineo Skin Closure System. Silk is one of the oldest biomaterials to have been used in healthcare; however, its use in modern medicine is still an emerging area of research. Woven silk scaffolds have only been studied in 4 clinical trials, excluding this study, and in approximately only 200 patients as reported by the FDA (Supplemental Table 1). These scaffolds have never been studied for surgical-site wound coverage, despite the plethora of preclinical and clinical research assessing the efficacy and safety of silk in tissue engineering.^14-18^

Silkworm silk is made of 2 distinct proteins: fibroin and sericin. Fibroin contains 2 parallel fibers, a light chain and a heavy chain, which are encased by the glue-like sericin protein.^17^ Silk fibroin has become a prominent material in tissue engineering due to its regenerative properties, showing high biocompatibility, low immunogenicity, and improved cell proliferation and growth.^19^ It has been approved for a wide array of FDA applications and has been thoroughly studied for the last 2 decades.^14-18^ It is utilized in the production of hydrogels, sponges, and films to aid in the natural regeneration of damaged tissue.^18^ Common applications include the regeneration of bone materials, artificial corneas, vascular grafts, nerves, tendons, and ligaments.^16^ It has also been used as an internal mesh as the Seri Surgical Scaffold (Sofregen, Framingham, MA) and as a medical textile to treat atopic dermatitis in Dermasilk (Alpretec, San Donà de Piave, Italy). Silk fibroin is also one of the strongest fibers found in nature (5-17 GPa), is elastic, extremely light, and is able to tolerate terminal sterilization with ethylene oxide for 24 h without diminution of its wound-healing properties.^20^

Silk fibroin is typically isolated from the globular sericin protein. Multiple studies on virgin sericin-containing silk suggest that the combination of sericin and fibroin induces an inflammatory response due to its allergenicity and immunogenicity.^21-25^ A growing number of studies have recently found that stand alone sericin is an antioxidant, antibacterial, and anticoagulase protein that can also promote cell differentiation and growth.^26-30^ Fibroin, however, remains the prominent structural component of natural silkworm silk, and in order to reduce the inflammatory cascade, sericin removal is standard for medical applications using silk. We believe that by utilizing a sericin-free silk bioprotein dressing, the high biocompatibility and increased regenerative attributes of silk fibroin will decrease the incidence of MARSI in surgically closed wounds.

## METHODS

### Study Design

A randomized, single-blinded, IRB approved (Western IRB, Puyallup, WA), prospective superiority clinical trial (clinicaltrials.gov NCT05508945) was conducted to compare the Dermabond Prineo skin closure system to a silk bioprotein wound dressing. The date range of the study was between May 11, 2022 and September 30, 2022. Twenty-five patients consented to the study and underwent abdominoplasty, belt lipectomy, mastopexy, or reduction mammaplasty procedures. After the incisions were closed, a 1:1 computer randomized generator was used to select the side for the application of the experimental silk dressing to control for surgeon operational bias. Sutured surgical incisions were dressed with the Dermabond Prineo skin closure system on the ipsilateral side and a silk bioprotein wound dressing on the contralateral side and thus each patient served as their own internal control. For bilateral operations, including reduction mammaplasty and mastopexy procedures, 1 breast was dressed with the Dermabond Prineo skin closure system, and the other breast was dressed with the silk dressing. For abdominoplasty and belt lipectomy procedures, half of the continuous incision starting at the midline was dressed using the Dermabond Prineo skin closure system, and the other half received the silk dressing. Patients were not told which dressing was experimental (silk) and which was the control (Dermabond Prineo). The physician and clinical trial staff refrained from using the dressing names and referred to each side as the left or right side of the patient to maintain patient blinding during follow-up appointments.

### Inclusion and Exclusion Criteria

All eligible patients signed written consent forms in accordance with the ethical standards of the Helsinki Declaration and satisfied the following inclusion criteria: ≥18 years of age, fluency in English, adequate cognitive ability to consent, and preoperative surgical clearance from a primary-care physician. Study participants were excluded for the following: history of an autoimmune disorder, diabetes, malignancy, tobacco use, and allergy to Dermabond 2-octyl cyanoacrylate adhesive, Prineo synthetic polyester mesh, or natural silk. Age, weight, height, BMI, and ethnicity/race were recorded.

### Interventions

Experimental silk wound dressings were created by laminating sterile Seri Scaffold (Sofregen Medical, Framingham, MA) 10 × 25 cm sheets with a pressure-sensitive acrylic adhesive (dermaFLEX; Flexcon, Spencer, MA) and a paper backing. Sheets were then cut into 2.5 × 25 cm strips, rolled into self-sealable autoclave pouches, and re-sterilized with ethylene oxide for 24 h. Two experimental dressings were made available per patient depending on the length of the incision. Control dressings consisted of 60 cm applicators of the Dermabond Prineo skin closure system (Ethicon, Raritan, NJ). Dermabond was applied using the adhesive pen applicator in the sterile packaging as instructed. The method utilized to apply the silk dressing and Dermabond Prineo is shown in the Video.

### Outcomes

The primary outcome was the incidence of ACD as indicated by a custom prepared investigator administered questionnaire to assess the occurrence of discomfort, itching/irritation, and rash. The questionnaire was given at clinical observation points on Days 1 to 3 and at Weeks 1, 2, 4, and 6. Patients were prompted to self-report symptoms on the questionnaire on a scale of 0 to 10, where 0 was no discomfort or itching and 10 was severe or intolerable skin discomfort or itching. Photographs were taken at each visit, and investigator observations were made about the presence of rash or erythema on a scale of 0 to 10, where 0 was the absence of rash or erythema and 10 was severe. Scores were categorized as 0 to 3 indicating a mild to no reaction, 4 to 6 indicating a moderate reaction, and 7 to 10 indicating a severe reaction. Parameters for the patient administered questionnaire and the assessment of rash/erythema were created with the guidance of the Joint Task Force on Practice Parameters for Allergy and Immunology for Contact Dermatitis.^31^

Secondary outcomes are as follows. Postoperative incisional separation or wound dehiscence, the need for topical or oral steroids to treat complications of ACD and the need for oral or intravenous antibiotics to treat cellulitis or presumptive infection. Time needed for removal was recorded as the time in seconds the physician took to remove each wound dressing. The time was recorded using a phone timer by trained clinical trial personnel. Detachol adhesive remover (Ferndale Laboratories, Ferndale, MI) was used to remove any excess adhesive remaining on the skin on either the experimental or control side. In accordance with manufacturer recommendations on the control side, the Dermabond Prineo skin closure system was removed 14 days after the surgical procedure. The experimental silk wound dressing was also removed at Day 14 post procedure. Premature detachment of the dressing prior to the anticipated removal on postoperative Day 14 was recorded for either side. Bathing routines for each patient were also recorded, including the type of shampoo or soap utilized. Early removal of the surgical-site dressing prior to postoperative Day 14 was performed by the treating physician with the sign of moderate-to-severe ACD, including rash, infection, or excess pain. If complications were localized to one side of the incision or 1 breast, the wound dressing on the unaffected side was not removed until postoperative Day 14 as scheduled. The data were collected and managed in an Excel spreadsheet (Microsoft Corp, Redmond, WA).

### Statistical Analysis

The sample size calculation was justified for 25 patients. As each patient received both treatments, the calculated sample size was 50 (25 silk and 25 Dermabond Prineo). This was based on the anticipated incidence of MARSI of 28% for the Dermabond Prineo control side and an incidence of 1% for the silk wound dressing with a power of 80% and an alpha level of 0.05. The incidence of MARSI for the Dermabond Prineo control side was based on prior literature to select an anticipated value of reaction.^1,32,33^ There is no reported literature for the use of a silk wound dressing, as this is the first indication of use. Based on the experience of the authors, an estimated value for the experimental silk dressing was determined.

Statistical analyses were performed using SPSS (SPSS Inc., Chicago, IL) software version 16.0. The null hypothesis (Ho) is that there is no difference in the outcomes of the silk bioprotein dressing to the outcomes of Dermabond Prineo. The alternative hypothesis (Ha) is that the performance of the silk dressing is different than the performance of Dermabond Prineo in wound management of breast lift/reduction and abdominoplasty/body lift patients.

When paired *t*-tests were performed, the underlying data were normally distributed and this was confirmed by the Kolmogorov–Smirnov and Shapiro–Wilk tests of normality as well as for the grams of tissue removed. For the time needed to remove Dermabond Prineo vs silk, the data were not normally distributed and were thus analyzed with a Wilcoxon Signed Rank test and noted to be statistically significant with a 2-tailed *P*-value .000. A mixed model for statistical analysis of longitudinal data was not used. Data for the time needed to remove surgical dressings are only a 1-time event, and data are collected only once in this entry. Similarly, infections and other reactions were not anticipated to happen at multiple time points, but only once if they occur.

The primary endpoint in the determination of superiority was ACD which included rash, itch, and discomfort scores. To calculate the overall rate of ACD, the highest numerical score at follow-up assessment was utilized.

## RESULTS

The demographic data of the patients treated are provided in Table 1. The average age of participants was 40 with a range between 19 and 66 years of age. All 25 participants were biologically females and 2 patients identified as transgender males.

**Table 1.** Type of Procedure and Demographic Data of the Study Group (25 Patients)

Demographics	Mean	Median	Mode	Minimum	Maximum
Age	40.7	38	29	19	66
BMI	27.8	25.9	25.6	18.4	39.7
Weight (lbs)	164.5	160	140	103	237
Procedure				Frequency	%
Abdominoplasty				7	28
Body lift				1	4
Breast reduction				14	56
Mastopexy				3	12

In total, 64% (16/25) of patients characterized the severity of discomfort as 4 or higher on the Dermabond Prineo control side and only 4% (1/25) for the silk-dressing side (*P* < .001). Statistical significance was calculated per follow-up appointment and is shown in Table 2. Thirty-six percent (9/25) had a discomfort level reported at a score of 7 or higher on the Dermabond Prineo side, and the silk side had no severe reactions (*P* < .001). Patients experienced statistically significant decrease in discomfort on surgical sites treated with the silk wound dressing compared to sites treated with Dermabond Prineo. The highest significance was seen at 2 weeks postoperatively and the differences between the dressings subsided in the later follow-up weeks (Table 2).

**Table 2.** Frequency of Discomfort, Itching, and Rash

Follow-up time	Type of discomfort	Frequency of moderate-to-severe reaction (%)	*P*-value^a^	Frequency of any reaction (%)^b^	*P*-value^a^
First follow-up visit (1-4 days after procedure, *n* = 25)	Silk skin discomfort	0 (0%)	.016*	0 (0%)	.004*
	Prineo skin discomfort	7 (28%)		9 (36%)	
	Silk itching	0 (0%)	.5	0 (0%)	.016*
	Prineo itching	2 (8%)		7 (28%)	
	Silk rash	0 (0%)	.5	0 (0%)	.004*
	Prineo rash	2 (8%)		9 (36%)	
Second follow-up visit (1 week after procedure, *n* = 25)	Silk skin discomfort	0 (0%)	.063	1 (4%)	.004*
	Prineo skin discomfort	5 (20%)		10 (40%)	
	Silk itching	1 (4%)	.5	2 (8%)	.031*
	Prineo itching	3 (12%)		8 (32%)	
	Silk rash	0 (0%)	.25	0 (0%)	.063*
	Prineo rash	3 (12%)		5 (20%)	
Third follow-up visit (2 weeks after procedure, *n* = 25)	Silk skin discomfort	1 (4%)	.016*	2 (8%)	.002*
	Prineo skin discomfort	8 (32%)		12 (48%)	
	Silk itching	1 (4%)	.125	2 (8%)	.012*
	Prineo itching	5 (20%)		11 (44%)	
	Silk rash	0 (0%)	.002*	3 (12%)	.002*
	Prineo rash	10 (40%)		13 (52%)	
Fourth follow-up visit (4 weeks after procedure, *n* = 25)	Silk skin discomfort	0 (0%)	.031*	2 (8%)	.016*
	Prineo skin discomfort	6 (24%)		9 (36%)	
	Silk itching	1 (4%)	1	5 (20%)	.5
	Prineo itching	1 (4%)		7 (28%)	
	Silk rash (*n* = 24)	0 (0%)	1	1 (4.2%)	.063
	Prineo rash (*n* = 24)	1 (4%)		6 (25%)	
Fifth follow-up visit (6-8 weeks after procedure, *n* = 25)	Silk skin discomfort	0 (0%)	1	0 (0%)	.5
	Prineo skin discomfort	1 (4%)		2 (8%)	
	Silk itching	0 (0%)	—^c^	1 (4%)	.25
	Prineo itching	0 (0%)		4 (16%)	
	Silk rash (*n* = 24)	0 (0%)	1	0 (0%)	.25
	Prineo rash (*n* = 24)	1 (4.2%)		3 (12.5%)	
Overall (the highest scores recorded in all 5 visits, *n* = 25)	Silk skin discomfort	1 (4%)	<.001*	4 (16%)	<.001*
	Prineo skin discomfort	16 (64%)		18 (72%)	
	Silk itching	2 (8%)	.07	7 (28%)	<.001*
	Prineo itching	8 (32%)		19 (76%)	
	Silk rash (*n* = 24)	0 (0%)	<.001*	5 (20%)	<.001*
	Prineo rash (*n* = 24)	13 (52%)		19 (76%)	

^a^Related samples McNemar test. Exact significance is displayed. ^b^Frequency of any recorded score except “0” (No reactions excluded). ^c^The test could not be executed. *Statistically significant *P*-values.

**Figure 2.**
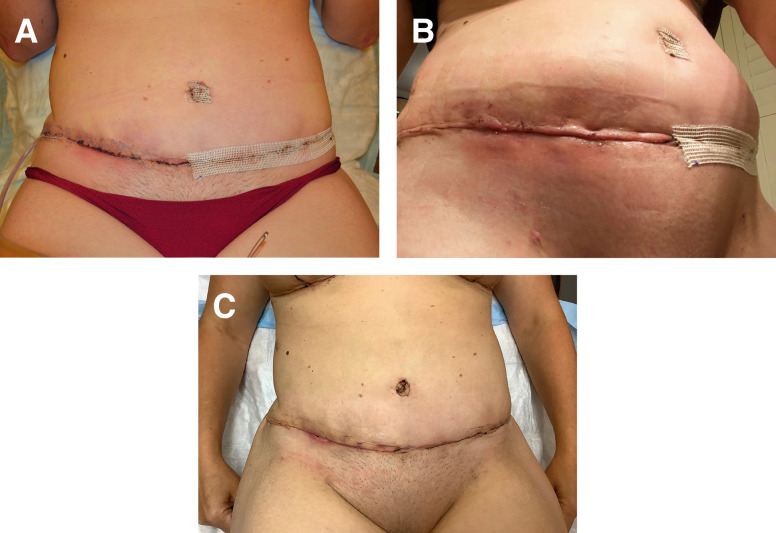
Photographs of a 38-year-old female on Days (A) 9, (B) 11, and (C) 14 status post abdominoplasty demonstrating the development of a pruritic rash and surgical-site infection on the Dermabond Prineo (Ethicon, Inc.) treated side of the abdomen. (A) The patient was seen on postoperative Day 9 with pain and erythema on the right side where Dermabond Prineo was applied. The Dermabond Prineo dressing was removed and the patient was prescribed topical and oral antibiotics. (B) The patient submitted a photograph on Day 11, demonstrating cellulitis on the right side where Dermabond Prineo was applied. (C) The patient was seen on Day 14 with evidence of partial wound dehiscence, rash, and discomfort localized to the patient's right side where Dermabond Prineo (Ethicon, Inc.) had been applied. The patient's left side dressed with the silk wound dressing experienced no complications.

**Figure 3.**
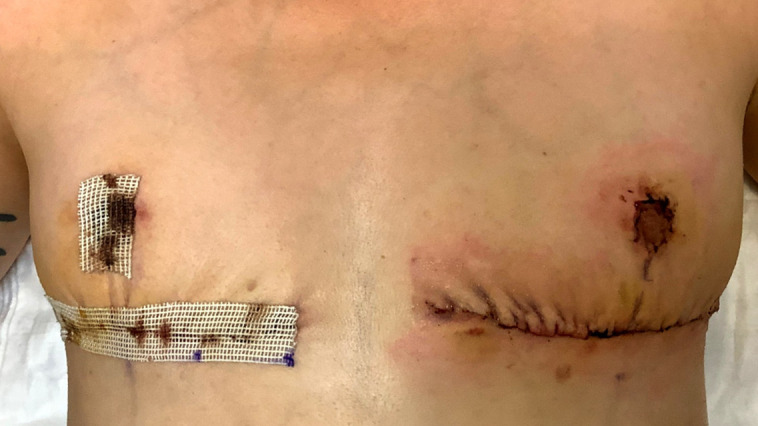
Photograph of a patient who developed moderate-to-severe allergic contact dermatitis to Dermabond Prineo (Ethicon, Inc.) localized to the patient's left side of the chest. The patient is a 29-year-old transgender male on postoperative Day 4 status postreduction mammaplasty using the Passot technique. The patient's right chest dressed with the experimental silk wound dressing was unaffected. The Dermabond Prineo dressing on the patient's left was prematurely removed, and the patient was treated with topical steroids for 7 days.

**Figure 4.**
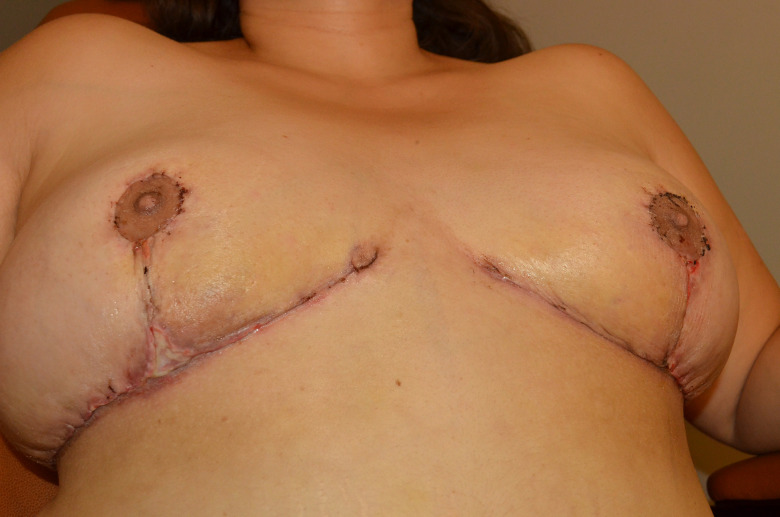
Photograph of a 36-year-old female patient on day 14 status post reduction mammaplasty. Patient presented with triple point separation on her right breast dressed with the Dermabond Prineo Skin Closure System (Ethicon, Inc.; Sumerville, NJ). The patient’s left breast was dressed with the silk wound dressing.

**Figure 5.**
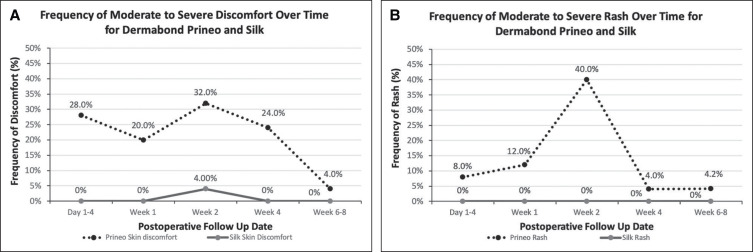
Frequency of moderate-to-severe discomfort and rash over time for Dermabond Prineo (Ethicon, Inc.) and silk wound dressings. (A) Demonstrates the percentage of patients who experienced discomfort of 4 or higher over the 5 follow-up appointments for both dressings. Discomfort levels to Dermabond Prineo were highest in the first 4 weeks after the procedure followed by a decrease. Little to no discomfort was seen on the side with the silk dressing. (B) Demonstrates the percentage of patients who experienced rash or erythema of 4 or higher over the 5 follow-up appointments. The highest frequency of rash was seen in Week 2 to Dermabond Prineo and subsided in Weeks 4 to 6. No rash was seen on the side with the silk dressing.

Rash and erythema scores were identified by the physician using the same score distribution. Fifty-two percent (13/25) had a visible rash of 4 or higher on the Dermabond Prineo side of their incision and 0% (0/25) had a rash on the silk side (*P* < .001). Six of these patients (6/25, 24%) had a severe pruritic rash of 7 or higher to Dermabond Prineo which also included erythema and required premature removal of the dressing (Figures 2-4). No patients had a severe rash on the silk-dressing side (*P* < .001). Statistical significance for rash scores is also represented in Table 2 with the greatest difference occurring at the 2-week follow-up appointment, decreasing after 4 weeks. Comparison between the frequency of discomfort and rash with Dermabond Prineo and the silk dressing are shown in Figure 5.

In total, 52% (13/25) of sites treated with Dermabond Prineo required pharmaceutical intervention (topical/oral steroids or oral/intravenous antibiotics) and 0% (0/25) required pharmaceutical treatment for the silk-dressing side (*P* < .001). The complications that required intervention included moderate-to-severe rash, pruritus as well as localized SSIs. Types of treatment for MARSI to Dermabond Prineo are shown in Supplemental Table 2.

The mean time to remove the silk dressing was 47 s and 1 min 39 s for the removal of Dermabond Prineo. This yielded statistically significantly faster times to remove the silk dressing compared to Dermabond Prineo with *P* < .001. For all breast reduction patients, the median weight of tissue removed per breast was 618 g and was similar for the silk-dressing side and the Dermabond Prineo side *P* = .96.

## DISCUSSION

### Assessment of Wound Dressings

This study has revealed an alarming range of MARSI-associated complications to the Dermabond Prineo skin closure system with ACD being the most significant, causing erythema, pruritus, and discomfort. In our study, 52% (13/25) of patients had a moderate rash to Dermabond Prineo and 24% (6/25) of those patients had a severe pruritic rash requiring pharmaceutical intervention. No patients presented with ACD to the silk dressing (*P* < .001). In comparison to the reported medical literature, rates of ACD to the Dermabond Prineo skin closure system vary significantly between studies. In 2020, a Therapeutic Level IV study of 100 patients by Nigro et al found the rate of ACD to Dermabond to be 14% (14/100) in breast surgeries.^34^ Nakagawa et al in 2020 found the rate of ACD to Dermabond to be 7% (7/100) in breast reconstruction cases.^35^ In a Level IV retrospective case series in 2017, Chalmers et al showed the reported rates of ACD to be as low as 0.5% (29/6088) in patients who underwent orthopedic surgery.^11^ The results of this retrospective study represent how MARSI complications are significantly underreported in our healthcare system. The variability of results in medical literature can also be due to differences in surgical sites, study types, sample demographics, and ACD categorization criteria.

A study that sampled approximately 20% of all US hospitalizations from 2002 to 2012 found a statistically significant association between adults with contact dermatitis and the rate of bacterial-site infections.^36^ This study also found that if treatment of ACD is postponed due to a delay in diagnosis, the intensity of ACD will increase and secondary complications such as SSIs will have a higher rate of occurrence.^36,37^ Early incidence of ACD should be treated with pharmaceutical intervention to reduce the incidence of SSIs, wound dehiscence, and other secondary complications as a result of ACD. An SSI or wound separation may also imperil an underlying prosthetic device (such as a breast implant) by potentiating higher rates of capsular contracture via biofilm formation or periprosthetic infection requiring explantation.^38^

Furthermore, unlike the Dermabond Prineo system which uses a hard-setting adhesive that limits oxygen permeability, the silk bioprotein dressing allows for oxygen enrichment of the healing surgical site to aid with tissue regeneration.^39^ The Dermabond 2-octyl cyanoacrylate adhesive polymerizes to form a hard barrier on the surface of the wound which may affect the permeability of oxygen and be an additional cause for resulting complications. The polymerization of Dermabond which seeps into the microscopic gap between wound edges may also affect wound healing by preventing true edge-to-edge contact. The woven nature of the silk mesh and its natural characteristics also allow for moisture vapor and exudate transmission from the wound site which decreases the risk of infection.^40^

**Figure 6.**
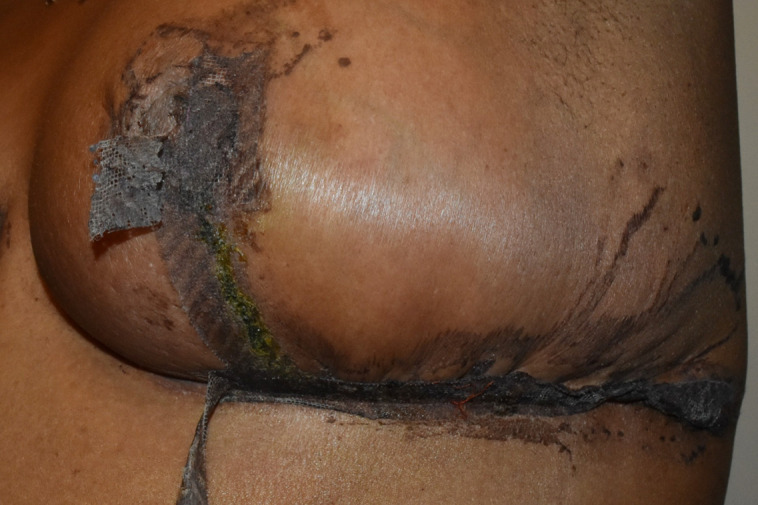
Photograph of black tar-like residue surrounding the patient's incision as a result of the Dermabond Prineo (Ethicon, Inc.) skin closure system. The patient is a 58-year-old female on postoperative Day 18 status postreduction mammaplasty. This patient required the application of Detachol adhesive remover (Ferndale Laboratories) in order to dissolve the hardened adhesive surrounding the incision. The Prineo mesh was also detaching from the areola and the inframammary fold preapplication of Detachol.

Our analysis of removal times also found that the silk dressing took less time to remove compared Dermabond Prineo. Moreover, Dermabond Prineo frequently required the use of an adhesive remover to dissolve the gummy black tar-like adhesive (Figure 6). Patients noted that the removal of the silk wound dressing was more comfortable than the removal of Dermabond Prineo. This was reflected by skin tears and pain during the removal of the Dermabond Prineo dressing and no injury during the removal of the silk wound dressing (Supplemental Figure 3). Both dressings remained attached during bathing routines.

### Economic Impact of Medical Adhesive-Related Skin Injuries

ACD is typically treated with topical steroid ointments and when there is a superimposed infection, with oral or IV antibiotics which can range from $20US to $800US per patient, depending on the severity of the reaction. The cost of treatment for poor scar outcomes including laser treatments or surgical revisions range from $400US to $5000US per patient.^41^ SSIs may require weeks or months of wound treatment and antibiotics, and may cause damage to underlying structures including the exposure of implanted materials.^42,43^ The incidence of SSIs following breast procedures including mastectomies, breast reconstruction, and breast augmentation has been estimated to be 5.3% with an average financial cost of $4091US per patient.^44^ Given the 700,000 cosmetic and reconstructive breast surgeries performed each year in the United States, there may be a cost of up to $150 million US per year due to SSIs for breast surgery alone. It is estimated that SSIs for all surgical procedures cost the United States healthcare system $3.5 billion US to $10 billion US annually.^45^ These costs encompass increased hospitalizations, the need for reoperation, the use of medical supplies, diagnostic tests, and clinician fees.^42^ Decreasing the incidence of MARSI can play a significant role in reducing the rate of SSIs, improving the patient care experience and improving access to healthcare in a less burdened system. The cost of Dermabond Prineo varies based on the contract and can average from $160US to $210US per 22 cm unit. We believe that a silk wound dressing can be competitively priced with a reduced adverse event profile.

### Regulatory Status and Future Research

We propose that a future silk wound dressing could be designated in the United States as an FDA Class 1, 510 (k) exempt, device with the product code KGX (tape and bandage, adhesive). This classification would establish a clear and safe regulatory pathway, enabling the product to be utilized in a variety of surgical settings. Future research that focuses on the use of hypoallergenic silk in medical applications will aid in the understanding of the biological rationale and mechanisms by which silk fibroin promotes wound healing. Although studies have identified the regenerative properties of fibroin in tissue engineering, few have analyzed its use in the context of a topical wound dressing. Future studies should also analyze the rates of MARSI for other wound dressings, surgical adhesives, and paper tapes.

### Limitations

The primary limitation of this study was in the relatively small sample size of 25 patients. Although each patient received both interventions leading to a total of 50 sites (25 Silk and 25 Dermabond Prineo), we believe that an increased sample size would have allowed for better statistical determination for secondary outcomes such as mechanical injury due to skin tears. As the surgical sites tested were paired, only 2 products could be compared with one another, and this prevented the additional testing of other common wound closures, such as adhesive paper tapes. Finally, though patients in the study were blinded as to which side was treated with Dermabond Prineo and which side received the silk dressing, it must be noted that both dressings had visual and textured differences and might have been discerned.

## CONCLUSIONS

This study is the first randomized controlled trial to assess the superiority of a woven silk bioprotein wound dressing compared to the Dermabond Prineo skin closure system. The advantages of a silk mesh laminated with a pressure-sensitive acrylic adhesive include ease of application and removal, resistance to detachment during normal postoperative bathing routines, the ability to be applied over irregular surfaces, and a low incidence of ACD or adverse mechanical injury. We postulate that the widespread adoption of a hypoallergenic silk bioprotein wound dressing has the potential to decrease the financial burden MARSI causes the healthcare system while improving the overall patient postoperative experience.

## Supplementary Material

ojad071_Supplementary_Data
