# Supplementary material for: Silk Bioprotein as a Novel Surgical-Site Wound Dressing: A Prospective, Randomized, Single-Blinded, Superiority Clinical Trial
Source: Aesthet Surg J Open Forum. 2023 Oct 20;5:ojad071. doi: 10.1093/asjof/ojad071 (PMC10603584; doi:10.1093/asjof/ojad071)
Supplement: ojad071_Supplementary_Data [file ojad071_Supplementary_Data.zip › 23-0063_Supplemental Table 2.docx]

**Supplementary Table 2:** Frequency and Type of Treatment

| Type of treatment needed | Frequency (%) |
| --- | --- |
| Both oral and topical antibiotic | 2 (8%) |
| Only oral antibiotic | 5 (20%) |
| Only topical steroid | 4 (16%) |
| Both topical steroid and antibiotics | 2 (8%) |
| Any pharmaceutical intervention | 13 (52%) |
| No treatment | 12 (48%) |
