# Supplementary material for: Silk Bioprotein as a Novel Surgical-Site Wound Dressing: A Prospective, Randomized, Single-Blinded, Superiority Clinical Trial
Source: Aesthet Surg J Open Forum. 2023 Oct 20;5:ojad071. doi: 10.1093/asjof/ojad071 (PMC10603584; doi:10.1093/asjof/ojad071)
Supplement: ojad071_Supplementary_Data [file ojad071_Supplementary_Data.zip › 23-0063_Supplemental Table 1.docx]

**Supplementary Table 1:** Clinical Trials of Silk Fabric/Scaffold Reported by the FDA

| NCT Number | Title | State | Conditions | Primary investigators/  sponsors | N | Date |
| --- | --- | --- | --- | --- | --- | --- |
| [NCT05508945](https://clinicaltrials.gov/show/NCT05508945) | Silk Scaffold Surgical Incision Dressing | A-R | Surgical incision | M. Mark Mofid, MD  San Diego Skin, Inc. | 100 | Aug 2022 |
| [NCT02293798](http://clinicaltrials.gov/show/NCT02293798) | Circumferential Periareolar Mastopexy Using SERI Surgical Scaffold | C | Mastopexy | M. Mark Mofid, MD Allergan Medical | 13 | Aug 2014 – Jun 2016 |
| [NCT02016612](http://clinicaltrials.gov/show/NCT02016612) | SERI Surgical Scaffold Support of the Lower Pole of the Breast | C | Recurrent ptosis of the breast | M. Mark Mofid, MD  Bradley P Bengtson, MD Allergan Medical | 76 | Oct 2013 – Nov 2016 |
| [NCT01914653](http://clinicaltrials.gov/show/NCT01914653) | SERI Surgical Scaffold Postmarket Study of Soft Tissue Support and Repair in Breast Reconstruction | C | Breast reconstruction | Sofregen Medical, Inc. | 17 | Jun 2013 – Mar 2016 |
| [NCT01389232](http://clinicaltrials.gov/show/NCT01389232) | The SERI Scaffold Use in Reconstruction Post Market Study for Tissue Support and Repair in Breast Reconstruction Surgery in Europe | C | Soft tissue support and repair | Sofregen Medical, Inc. | 104 | Jun 2011 – Feb 2015 |

List of 5 reported clinical trials (completed and recruiting) of silk fabric/scaffold as an implant or wound dressing. The state of the study recruitment is encoded as follows: A-R, active recruiting; C, recruiting completed; FDA, Food and Drug Administration; N, number of participants in each trial.
