# Supplementary figures and images for: Silk Bioprotein as a Novel Surgical-Site Wound Dressing: A Prospective, Randomized, Single-Blinded, Superiority Clinical Trial
Source: Aesthet Surg J Open Forum. 2023 Oct 20;5:ojad071. doi: 10.1093/asjof/ojad071 (PMC10603584; doi:10.1093/asjof/ojad071)

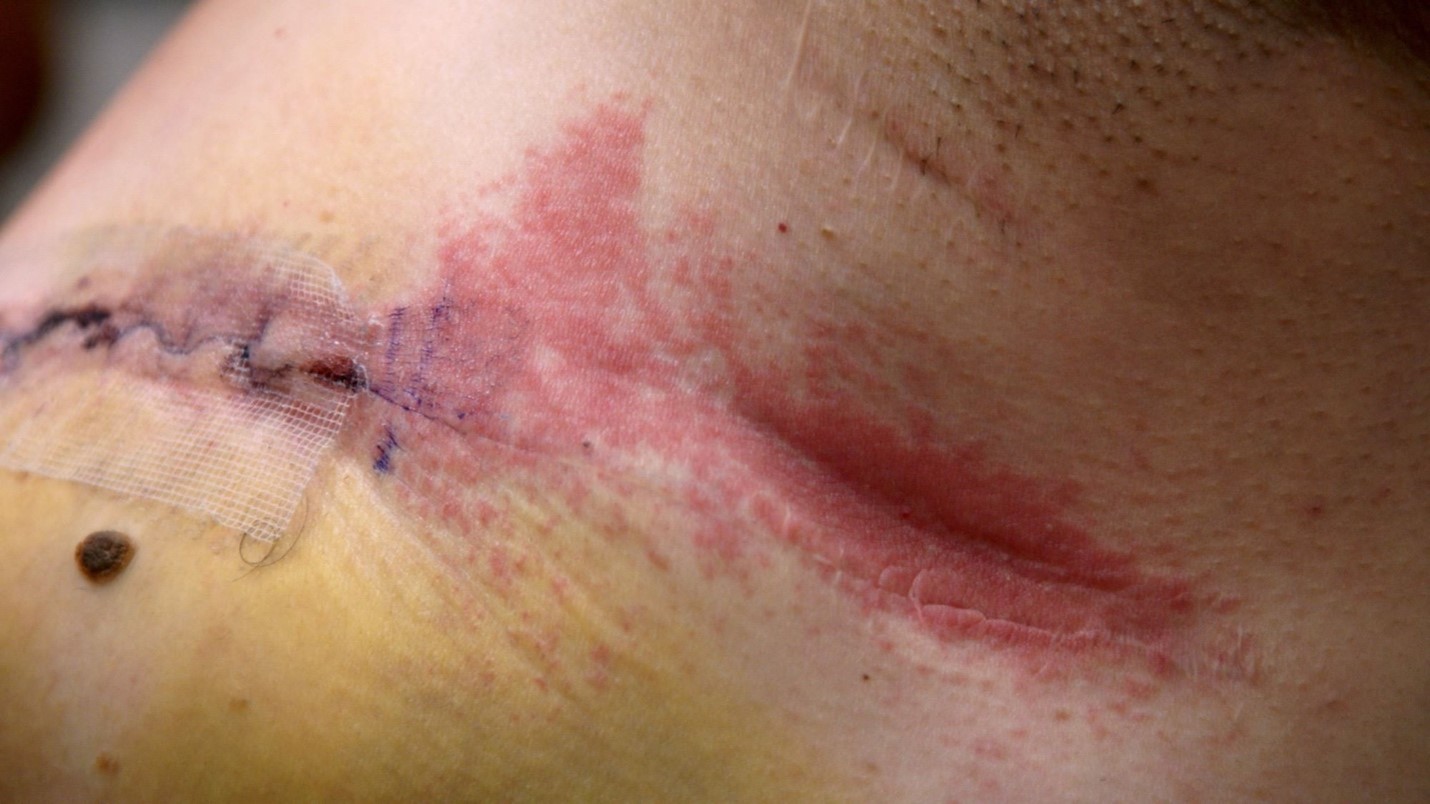

Supplement: ojad071_Supplementary_Data [file ojad071_Supplementary_Data.zip › 23-0063_Supplemental Figure 1.jpg]

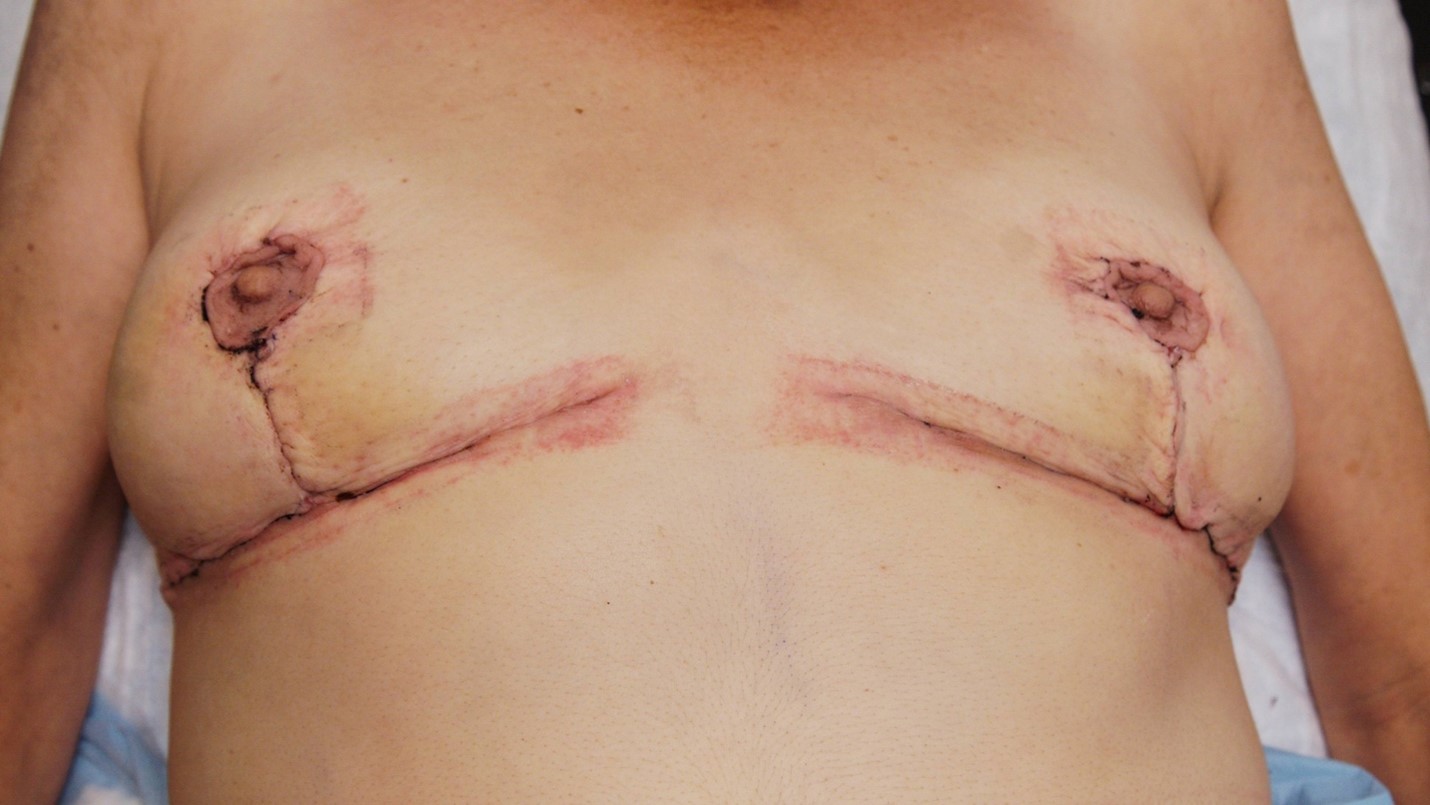

Supplement: ojad071_Supplementary_Data [file ojad071_Supplementary_Data.zip › 23-0063_Supplemental Figure 2.jpg]

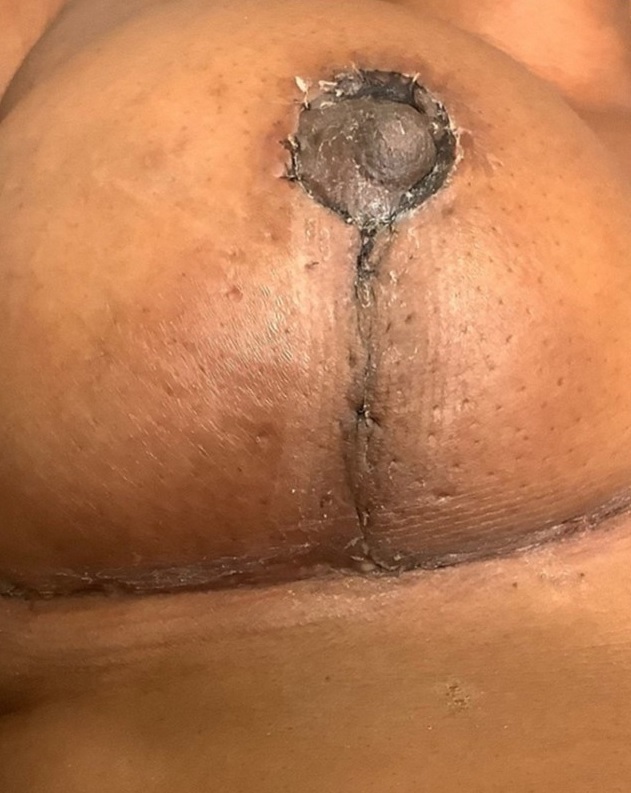

Supplement: ojad071_Supplementary_Data [file ojad071_Supplementary_Data.zip › 23-0063_Supplemental Figure 3A.jpg]

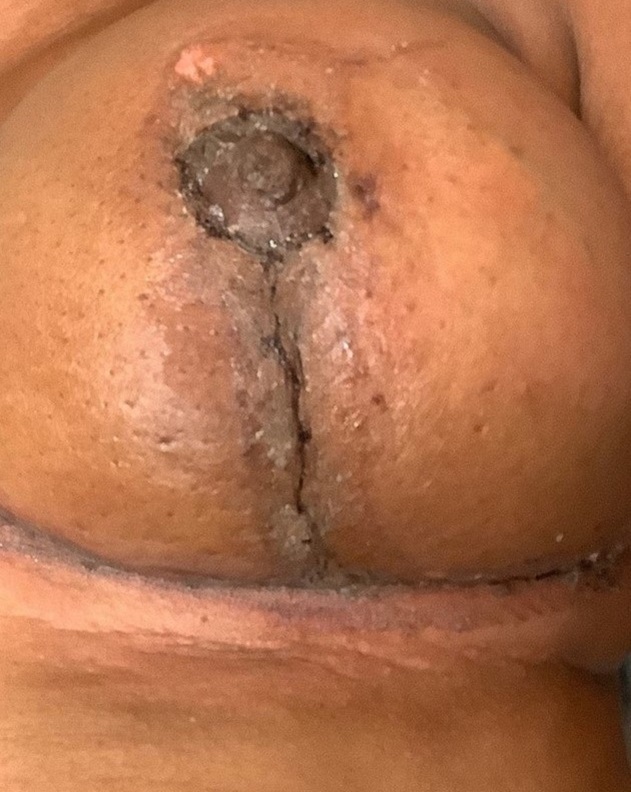

Supplement: ojad071_Supplementary_Data [file ojad071_Supplementary_Data.zip › 23-0063_Supplemental Figure 3B.jpg]
